# Subinguinal orchiectomy—A minimally invasive approach to open surgery

**DOI:** 10.1002/bco2.33

**Published:** 2020-08-13

**Authors:** Elliot Anderson, Claire Pascoe, Niranjan Sathianathen, Darren Katz, Declan Murphy, Nathan Lawrentschuk

**Affiliations:** ^1^ Department of Surgery Monash University Clayton VIC Australia; ^2^ Department of Urology Western Health Footscray VIC Australia; ^3^ Division of Cancer Surgery Peter MacCallum Cancer Centre East Melbourne VIC Australia; ^4^ Men’s Health Melbourne Melbourne VIC Australia; ^5^ Sir Peter MacCallum Department of Oncology University of Melbourne Parkville VIC Australia; ^6^ Department of Urology Royal Melbourne Hospital Melbourne VIC Australia; ^7^ Department of Surgery The University of Melbourne Melbourne VIC Australia; ^8^ EJ Whitten Centre for Prostate Cancer Research Richmond VIC Australia

**Keywords:** ilioinguinal nerve injury, modified orchidectomy, subinguinal, testicular cancer, testicular pain

## Abstract

**Objectives:**

To determine the rate of morbidity and assess the oncological outcomes for the subinguinal orchidectomy technique.

**Background:**

Radical inguinal orchiectomy is the definitive management for a testicular mass suspicious for malignancy. The standard approach involves the division of the spermatic cord at the internal inguinal ring. In addition to the morbidity of a significant incision through skin and fascia, a known complication is damage to the nerves within the canal leading to local hypoesthesia or persistent inguinal and scrotal neuralgia. The subinguinal orchiectomy technique avoids opening the inguinal canal by excising the spermatic cord at the external inguinal ring.

**Methods:**

Patient data from three urologists who routinely perform subinguinal orchiectomies for suspected testicular malignancy was collected. A retrospective analysis between March 2011 and March 2019 was undertaken evaluating demographic, clinical, and histological data points. Descriptive analysis of oncological and surgical outcomes of subinguinal orchiectomy for testicular mass was performed. Descriptive analysis of oncological and surgical outcomes of subinguinal orchiectomy for testicular mass was performed.

**Results:**

About 42 orchiectomies performed via the subinguinal approach were identified. The median age was 38 years (range 22‐72) and mean follow‐up time was 18.4 months (range 0.59‐61). Of the 38 patients with testicular cancer, histopathology showed 26 with pT1, 9 with pT2, and 3 with pT3 disease. Three patients had involvement of the cord, with one patient having a positive surgical margin secondary to venous invasion. No patients experienced neuropathic complications, hernia, or wound break down.

**Conclusion:**

These data suggest that subinguinal orchiectomy provides acceptable oncological outcomes, comparable to a traditional technique, and may decrease the risk of neuropathic injury and incisional/inguinal hernia. Further investigation with a larger, prospective series is required.

## 1 INTRODUCTION

The outcomes for testicular cancer have markedly improved over the last 30 years. Prior to 1970 the 5‐year survival rate was 64%, today the 5‐year survival rate is over 95%.^1^ This improvement has resulted from a better understanding of the natural history of testicular cancers, utilization of tumor markers, and the implementation of platinum‐based combination chemotherapy. Although classified as a rare tumor, testicular malignancy is the most common solid malignancy among young men aged 20‐39^2^ with approximately 850 new diagnoses estimated in Australia in 2019.^3^

The treatment of choice in men with suspected primary testicular cancer is radical inguinal orchiectomy.^4^ This enables histologic diagnosis and staging of the tumor type and provides local tumor control.^5^ Following their operation, men are placed on surveillance, receive chemoradiotherapy or proceed to retroperitoneal lymph node dissection (RPLND). The classic (high‐cord) radical inguinal orchiectomy technique is well described and involves a high ligation of the spermatic cord at the level of the internal ring with the delivery en bloc of the ipsilateral spermatic cord, testis, and surrounding tunica vaginalis.^6^, ^7^ Following an oblique incision parallel to the inguinal canal, the external inguinal ring is identified and the external oblique muscle is divided. This endangers the ilioinguinal nerve that must be identified and mobilized so as not to be damaged.

Although this procedure is reasonably straightforward with low reported morbidity, surgery of the scrotum is associated with a number of complications including postoperative wound hemorrhage, infection, inguinal hernia, and nerve injury.^8^, ^9^ Importantly, the ilioinguinal nerve, which arises from the lumbar plexus, supplies the inguinal canal and scrotum. If injured or compressed, then paresthesia or significant pain may develop postoperatively. Typically, these symptoms respond poorly to classic analgesia and may lead to repeated consultations and possible revision of the operating area. If the external oblique fascia is not effectively closed, a direct inguinal hernia may develop although this is a rare event (<1%).^8^

The subinguinal (low‐cord) orchiectomy approach avoids incision of the external oblique aponeurosis by ligating the spermatic cord at the level of the external inguinal ring and circumvents the path of the ilioinguinal nerve. Given that subinguinal orchiectomy is a simpler and less invasive technique compared to a traditional radical inguinal orchiectomy, we aim to establish its rate of morbidity and oncological outcomes to determine if it is a viable alternative to the standard approach.

## 2 METHODS

Between May 2011 and March 2019, cases of radical orchiectomy via a subinguinal approach were identified during a retrospective audit of theater records. The private data from a third Urologist (DK) who routinely utilizes the subinguinal approach was also included. Men were excluded from study if they underwent a radical orchiectomy for an indication other than suspected testicular cancer or if surgery was performed with an approach other than subinguinal.

**FIGURE 1:**
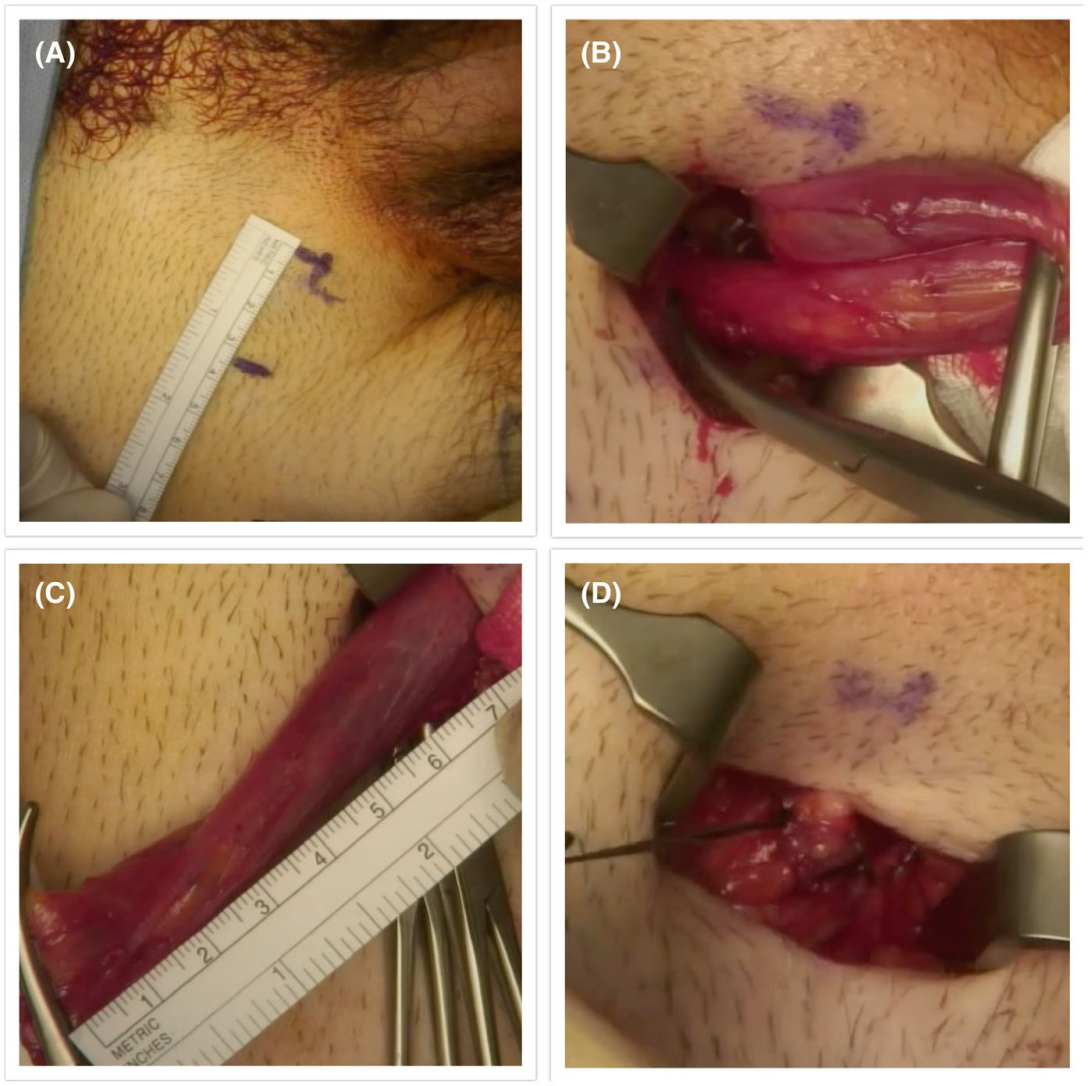
Technique for sub‐inguinal orchiectomy. A, External ring identified and marked. B, Spermatic cord identified and isolated. C, Length of spermatic cord removed with specimen. D, Testis delivered and dissected away from the gubernaculum, cord divided and transfixed using standard techniques

Using standard surgical techniques, a 3‐5 cm inguinal incision was made 2 cm superior and lateral to the pubic tubercle. Layers are then dissected to the external inguinal ring at which time the cord is clamped using two Roberts forceps. The cord is then mobilized and the testis delivered. It is then carefully dissected away from the gubernaculum. The cord is then divided and transfixed using standard techniques. After achieving hemostasis, the incision is closed in two layers (please see Figure 1). Local anesthetic was infiltrated into the wound at the end of the case. These were performed as day cases.

Data points relating to operative details, clinical, and pathological stage were extracted from clinical records. Histological variables included extent of tumor, intertubular germ cell neoplasia present, epididymis involvement, spermatic cord involvement, lymphovascular invasion, and margin status. Pathological staging of testicular tumors was conducted based on the American joint committee on cancer TNM classification. Patient complications were classified using the Clavien–Dindo system.^10^ Follow‐up was based on EAU guidelines,^11^ with the initial review within six weeks of the operation. At these appointments, men were asked about complications including neuropathy.

Statistical analysis was undertaken using Microsoft Excel and utilized summary/descriptive analysis only.

## 3 RESULTS

Overall 42 subinguinal orchiectomies were performed for suspected testicular cancer between 2011 and 2019. Two men were excluded from the study having undergone a subinguinal orchiectomy for an indication other than suspected malignancy (trauma, epididymo‐orchitis). The average age of men was 38 years' old (range 22‐72) and the most common indication for the operation was a suspicious mass identified on ultrasound. In total, 38 (90%) patients had a confirmed malignancy with n = 34 (89%) GCTs, sex cord stromal tumors n = 2 (5%), lymphoma n = 1 (3%), and sarcoma n = 1 (3%) (see Table 1). Mean total operating time was 48 minutes (range 20‐70 minutes) taking into account the majority of cases were undertaken at a training hospital with registrar involvement. Patients received standard postoperative care only requiring routine analgesia. The majority of men (35 (83%)) were discharged on the same day as their operation and of those who stayed overnight most remained for social reasons. The follow‐up duration for patients with confirmed testicular malignancy was 18.4 months (Range 0.59‐61).

**TABLE 1:** Demographics

Number of subinguinal orchiectomies performed = 42		
Age	Range	22‐72
	Median	38
	Mean	40
Site	Right	22
	Left	20
Indication	Suspicious clinical exam	17
	Ultrasound mass	22
	Undescended testicle	1
	Lymphoma	1 (postchemotherapy)
		1 (disease progression)
Testicular cancer (38)	Sex cord stromal tumor	2
	Sarcoma	1
	Lymphoma	1
	Testicular GCT	Seminoma 17
		NSGSCT 8
		Mixed 9
Benign		4

**TABLE 2:** American Joint Committee on Cancer TNM classification and histologic features of confirmed testicular cancer

pT0	4
pT1	26
pT2	9
pT3	3
pT4	0
cN0	36
cN1	3
cN2	3
cN3	0
M0	41
M1	1
*Confirmed testicular cancer = 38*	
Limited to testis	29
Intertubular germ cell neoplasia present	25
Epididymis involvement	4
Spermatic cord involvement	3
Lymphovascular invasion present	10
Margins clear	37

The microscopic pathological findings for the confirmed testicular cancers showed that 29 (76%) were limited to the testis, 25 (66%) had intertubular germ cell neoplasia present, 4 (11%) had epididymis involvement, and 10 (26%) showed lymphovascular invasion. Of note, three (7%) patients had tumor in the spermatic cord and one (2%) patient had a positive margin showing venous invasion. All patients that had nodal disease confirmed by pathology also had imaging, either computer tomography (CT) or positron emission tomography (PET), showing nodal disease (see Table 2).

Of the men with spermatic cord involvement, patient one (39 year old male) also had a positive surgical margin with venous invasion. His pathology showed mixed GCT (embryonal 95%, seminoma 5%) and he was staged as pT3cN2M0. He completed four rounds of chemotherapy and also underwent RPLND that did not reveal any malignant nodal disease. There was no reported difficulty removing the remaining spermatic cord at the time of RPLND. Following three years of surveillance, he remains in remission. Patient two (34 years old male) underwent left orchiectomy for stage one NSGCT, on a background of right orchiectomy (stage one seminoma) and left partial orchiectomy (stage one seminoma) four years earlier. He was found to have retroperitoneal lymph node disease at six months and achieved a complete response to three cycles of chemotherapy. He is currently disease free after 12 months of follow‐up. Patient three (33 years old male) was found to have a stage one seminoma and managed with adjuvant chemotherapy (single dose of carboplatin). He is recurrence free following five years of surveillance.

Intraoperatively, there was no reported difficulty enacting the subinguinal technique. No patients experienced neuropathic complications, hernia, or wound break down. Four patients developed postoperative seromas. Of these patients, three were successfully managed with percutaneous drainage (Clavien–Dindo IIIa) and one resolved with observation (Clavien–Dindo I).

## 4 DISCUSSION

The role for inguinal orchiectomy in cases of suspected testicular malignancy has long been the established standard of care for managing this disease. The scrotal approach to orchiectomy was found to be unacceptable due to the theoretical risk of spreading tumor cells to the additional lymphatic supply of the scrotum that drain to the superficial inguinal nodes. The historic rationale that a high ligation of the spermatic cord at the deep ring would better contain lymphatic spread of malignancy has not been well demonstrated, with the management paradigm for testicular cancer focusing on other factors such as radiographic and serological markers.^12^, ^13^

The testes and scrotum have a rich somatic supply, that arises from the L1‐2 and S2‐4 nerve roots and include the iliohypogastric, ilioinguinal, genitofemoral, and pudendal nerves.^14^ In addition, the testis has an extensive visceral nerve supply that may contribute to the development of phantom testis pain.^15^ The pathophysiology of scrotal pain is complicated and multifactorial, although entrapment or damage to one of the relevant nerves can result in chronic orchalgia.^16^ In particular, the ilioinguinal nerve and genital branch of the genitofemoral nerve that often share a close relationship within the inguinal canal are at risk of damage in operations where the inguinal canal is opened.^17^ Although the risk of morbidity associated with the standard orchiectomy technique is low, neuropathic complications can have a lasting effect on the typically young cohort of men that are affected by testicular cancer. Up to 25% of cancer testis survivors will develop phantom testis pain syndrome that may be permanent in some men.^15^ Chronic testicular pain has also been shown to significantly affect sexual health, with men reporting ejaculation disorders, erectile dysfunction and diminished enjoyment of orgasms following testicular cancer treatment.^18^ The subinguinal orchiectomy approach avoids opening the inguinal canal and as such preserves the path of the ilioinguinal nerve, reducing the risk of sequelae relating to iatrogenic injury. This analysis is analogous to varicocelectomy, where the subinguinal approach is associated with less postoperative pain and a lower risk of complications compared to an inguinal approach.^19^, ^20^, ^21^ This study found the subinguinal orchiectomy approach to be safe with no neuropathic complications identified.

The presence of spermatic cord invasion by testicular cancer is a significant pathological finding that conveys questionable clinical significance. Valdevenito et al reviewed the histopathology of 86 men diagnosed with pure seminoma, looking for pathological features that would indicate the presence of metastasis. They found using univariate analysis that tumor invasion of the base of the spermatic cord, among other risk factors, was significantly more common in patients with metastatic seminoma. This result was not validated by multivariate analysis and may have resulted from clinical staging bias. Given this limitation, a prospective study is needed to determine its clinical utility in predicting relapse.^22^ Additionally, recent changes in the latest 8^th^ edition of the American Joint Committee on Cancer (AJCC) staging manual now differentiate between discontinuous invasion of the spermatic cord from involved lymphovascular spaces and direct extension. Discontinuous invasion is classified as stage three, a worse prognostic indicator.^23^ Notably, in previous guidelines, the pathological assessment of an orchiectomy specimen had little impact on clinical staging, as pT1‐pT4 testicular tumors without evidence of nodal or metastatic spread were grouped as prognostic stage one. To evaluate the latest AJCC staging protocol, Sanfrancesco et al compared the risk of cancer recurrence between direct spermatic cord involvement and discontinuous invasion and found no statistical difference.^24^ Further to this, Haroon and colleagues analyzed the histopathology of 72 men with testicular malignancy that had undergone a radical inguinal orchiectomy and reviewed the relevance of spermatic cord invasion as an independent predictor for worse disease prognosis. They found that the spermatic cord was involved in three of the specimens and had no impact on the overall or five‐year survival.^25^

Another concern for oncological compromise when utilizing the subinguinal technique relates to the risk of local recurrence. In our study, all three men who had spermatic cord involvement were disease free following adjuvant chemotherapy and no patient in the study had local recurrence. This is a similar finding to Ashdown et al, who completed the largest comparison of high‐cord versus low‐cord orchiectomies. They found no difference between approaches for relapse or mortality for clinical stage 1 tumors. There was also no recorded local recurrence in the inguinal canal with either approach after more than 40 months of follow‐up.^26^ This highlights that although local recurrence within the inguinal canal is possible with the subinguinal approach, it is a low risk.

This study is subject to a number of limitations. Its retrospective nature impacts its level of evidence and prescribes a variable duration of follow‐up. In addition, this study is restricted by its small sample size and lack of comparative (standard inguinal orchiectomy approach) cohort.

## 5 CONCLUSION

Neuropathic complications can result following inguinal orchiectomy and are associated with marked morbidity that can have a lasting effect on a cohort of typically young men. By avoiding opening the inguinal canal, the subinguinal orchiectomy approach reduces the risk of iatrogenic nerve injury and its complications. In addition, it is a quicker and technically simpler procedure that also mitigates the risk of a postoperative inguinal hernia developing. These data suggest that subinguinal orchiectomy provides acceptable oncological outcomes, with spermatic cord involvement not impacting on survival. Further investigation with larger, prospective studies is required.

## CONFLICT OF INTEREST

None.
